# Expanding the reach: ethnobotanical knowledge and technological intensification in beekeeping among the Ogiek of the Mau Forest, Kenya

**DOI:** 10.1186/s13002-020-00409-w

**Published:** 2020-09-29

**Authors:** Dauro Mattia Zocchi, Gabriele Volpato, Duncan Chalo, Patrick Mutiso, Michele Filippo Fontefrancesco

**Affiliations:** 1 University of Gastronomic Sciences, Bra, Italy; 2 University of Nairobi, Nairobi, Kenya

**Keywords:** Honey production, Log hive, Modern hive, Melliferous plants, Livelihood change

## Abstract

**Background:**

Initiatives for beekeeping intensification across the tropics can foster production and income, but the changes triggered by the introduction of modern beehives might permeate traditional knowledge and practices in multiple ways, and as such should be investigated and understood. We conducted an ethnobotanical study in the Eastern part of the Mau Forest among Ogiek beekeepers who customarily practice forest beekeeping and who are involved in a project aimed at the modernization of their beekeeping activities. We aimed to document the beekeeping-associated ethnobotanical knowledge, exploring the relationships and complementarity between modern and traditional knowledge and practices.

**Methods:**

Field research was carried out through semi-structured interviews with 30 Ogiek beekeepers and 10 additional stakeholders. We collected ethnobotanical data about plants used for beekeeping purposes, and ethnographic information on traditional and modern beekeeping systems.

**Results:**

We report 66 plant species, distributed across 36 botanical families representing 58 genera, important as melliferous, for the construction and placing of hives, attracting bees, and harvesting and storing honey. *Dombeya torrida* (J.F.Gmel.) Bamps, *Juniperus procera Hochst*. ex Endl., and *Podocarpus latifolius* (Thunb.) R.Br. ex Mirb. are the species with the most mentions and the highest number of uses. Our study reveals that the Ogiek possess a detailed knowledge of the forest’s flora, its importance and uses and that this knowledge underpins beekeeping practices. Under the influence of external actors, the Ogiek have progressively adopted modern versus traditional log hives and moved beekeeping out of the forest into open areas of pastures and crop fields. Beekeepers are also experimenting with combinations of practices borrowed from modern and traditional beekeeping systems, particularly in the field of hive construction and in the criteria to set up apiaries.

**Conclusions:**

The study indicates a complementarity and an incipient hybridization of traditional and modern beekeeping, in a way that suggests that modern beehives are instrumental in expanding the reach of beekeeping into deforested and cultivated areas. The study also points to the existence of a rift in the effects of beekeeping intensification on the livelihoods of the Ogiek and on their relationship with the forest. We argue that this intensification might be improving the former but weakening the latter, carrying the associated risk of erosion of traditional forest-based ethnobotanical knowledge.

## Introduction

Beekeeping and honey hold a very important economic, social and cultural role for several ethnic groups and rural communities across the tropics [1, 2]. Considering the potential of beekeeping for income generation and poverty alleviation among rural dwellers, national governments, NGOs and international institutions have implemented apiculture development programs aimed at expanding the practice, modernizing bee management and increasing honey production [3, 4]. In the tropics, where forest ecosystems are critically threatened by human encroachment, traditional forest beekeeping is seen as a sustainable practice, whose maintenance and promotion can contribute to the conservation of forests and associated biodiversity, as well as to the resilience of local communities [5, 6]. The strategy at stake in these initiatives is multi-level and aimed at the promotion of production in three main directions: *intensification* of honey production in terms of quantity per beehive/beekeeper (e.g. through the introduction of modern beehives and techniques, training and extension programs), product *valorisation* (e.g. improvement of marketability) and *expansion* in terms of the number of beekeepers and land used [3, 7, 8]. Initiatives of this kind have mainly focused on the replacement of traditional hives with the introduction of modern hives, based on the notion that the latter have higher yields than the former [9, 10].

In Kenya, beekeeping plays a fundamental role in communities living in forested as well as in arid and semi-arid areas, with most beekeepers relying on indigenous and traditional knowledge and skills [1, 11, 12]. The practice is carried out in small-scale extensive systems, largely using traditional log hives scattered over large areas of forest and savanna [13]. The promotion of beekeeping has been an important element in the rural development policies of the Kenyan government and international agencies [14], aiming to increase productivity in the honey sector and facilitate the marketing of bee products by setting up producers’ cooperatives. Several initiatives promoted a shift from traditional log hives to modern hives (first Kenyan Top Bar hives and then, since the early 2000s, Langstroth hives), as the former were not deemed suitable to meet market demand for honey in terms of quality and quantity [14, 15].

Despite the great efforts made to modernize the beekeeping sector, the claimed production potential is still underdeveloped and the contribution of beekeeping to the improvement of rural livelihoods remains unexpressed [12]. Previous studies have reported that the adoption of modern hives has been slow as a result of their high cost for rural dwellers, the lack of training on how to manage them and their scarce adaptability to forest ecologies [16, 17]. Although some scholars have addressed drivers and variables connected to the adoption of modern beekeeping systems [13, 14], there is still little information about the actual impacts of this innovation on traditional beekeeping-related ethnobotanical knowledge and practices and on the ways of living in the environment by local communities [18, 19]. Other studies conducted in Africa [6, 20] and elsewhere [21, 22] have highlighted the difficulties for intervention programs to strike a balance between fostering economic empowerment while promoting environmental conservation and supporting cultural diversity. These aspects are of particular relevance in those contexts where beekeeping and honey have already been part of traditional livelihood strategies, as technological innovations and production intensification can trigger changes that feedback on the complex and dynamic relationships between humans and the surrounding environment, and on the knowledge that underpins them [23, 24]. As such, these changes should be investigated, understood and embedded into development initiatives [25].

In order to investigate these issues, this study steps away from a representation that reproduces an antithesis between traditional and modern knowledge [26–28], and instead focuses on the relationships, tensions and complementarity of different knowledge and practices and the ways in which they are employed in beekeeping strategies. Specifically, we explore the ways in which the introduction of modern hives and the socioeconomic and environmental changes that occurred in the last few decades have transformed the beekeeping-associated ethnobotanical knowledge and practices of the Ogiek of the Mau Forest in Kenya. The specific objectives were (1) to document the social and spatial organization of beekeeping in the different ecological zones of the study area, (2) to inventory the diversity of plants used in beekeeping and associated knowledge and (3) to explore differences between traditional and modern beekeeping with regard to the knowledge and use of melliferous plants and to the flora used for the construction of beehives and for harvesting and storing honey. To this end, we carried out fieldwork in the Eastern part of the Mau Forest, focusing on beekeeping activities in a context where local beekeepers are involved in a project aimed at promoting honey production through the modernization of beekeeping practices. Although scholars have carried out ethnobotanical studies among the Ogiek in the past few decades [29–31], beekeeping-associated knowledge has been scarcely investigated and the interplay between this knowledge, technological intensification and beekeeping has gone unacknowledged.

## Background

The Mau Forest, located in the Rift Valley of Kenya, is one of the largest remaining closed-canopy montane forests in Eastern Africa and one of the most important honey-producing regions of Kenya [13, 32, 33]. During the last century, the Mau Forest has been affected by anthropogenic activities (e.g. population growth, agricultural expansion, land privatization, logging) that have reduced primary forest cover [34, 35], as much as 850 km^2^ or 43.5% in the last 40 years in its Eastern part [36].

The Mau Forest has traditionally been inhabited by the Ogiek, a hunter-gatherer group belonging to the Nilotic ethnic mosaic [37, 38]. The Mau Forest was the place where the Ogiek carried out social and cultural practices, where they procured the food they needed and thus where their identity was rooted. Given its importance as a home and provider, the forest was managed in accordance to a set of ethical principles and customary norms (e.g. of access and use of specific areas and species) that defined Ogiek-forest relations. The Ogiek customarily relied on the forest for subsistence, practising mobile beekeeping and hunting as main food procurement activities [37, 39]. Each Ogiek clan had exclusive rights to transects of land, called *konoito*, which comprised ecological zones located at different altitudes in the forest [38, 40]. Thousands of log hives were spread across the forest, located high up in specific trees, and honey production relied on the spontaneous occupation of the hives by swarms of bees tracking flowering plants in different seasons in different forest habitats [40].

Honey and beekeeping were central to the economic, social, cultural and religious life of the Ogiek [37–40]. Beekeeping was a male-dominated activity, with men in charge of log hive construction and placement, as well as honey harvesting. Women helped to carry honey from the forest to the homestead and to move the hives from one location to another inside the forest [37, 39]. Honey from specific plants was used for brewing honey mead (*rotinik*), as a natural preservative for wild meat and also as an ingredient for medicinal preparations. Honey and its derivatives also played a central role in traditional rituals, e.g. in circumcision ceremonies and dowry payments, and as items of local exchange with neighbouring communities, such as the Maasai [38, 39].

However, during British colonial rule and later after Kenyan independence, the relationship between the Ogiek and the forest changed with the creation of natural reserves and the encroachment of economic and extractive activities, such as small-scale agriculture, tea cultivation, and exotic tree plantations [41]. Following independence in 1963, the Kenyan government accused the Ogiek of being illegal squatters in the Mau Forest [41, 42]. The Ogiek were progressively evicted and resettled at mid-altitude, where they began growing crops and rearing livestock [43]. Eventually, in 1997, the Ogiek filed a constitutional case against the Kenyan government and in 2017 the African Court on Human Rights and Peoples’ Rights in Arusha recognized the Mau Forest as the ancestral home of the Ogiek and their role in safeguarding it [44].

Nowadays, there are between 20,000 and 60,000 Ogiek living in Kenya, most of them in the Mau Forest and its surrounding area and around Mount Elgon, near the Ugandan border [45]. They can carry out productive activities in the forest as long as they belong to government-organised Community Forest Associations [46, 47]. Some Ogiek households have further joined self-help groups and community-based organizations that are involved in agricultural activities, reforestation programs and beekeeping, often receiving technical and financial aid from county extension offices, NGOs and other international organizations [48].

However, a century of displacement, replacement and negotiation of their relationship with the forest has led the Ogiek to a more sedentary life, thus reducing their reliance on the forest itself [29, 48]. At the same time, they shifted from a subsistence model based on hunting and gathering to a system that integrates traditional livelihood activities with cash crop farming, which is influenced by external actors and introduced technologies, ideologies and social relationships. It is within this socioeconomic and ecological framework that current beekeeping practices among the Ogiek should be understood.

## Methods

### Study area

**Fig. 1:**
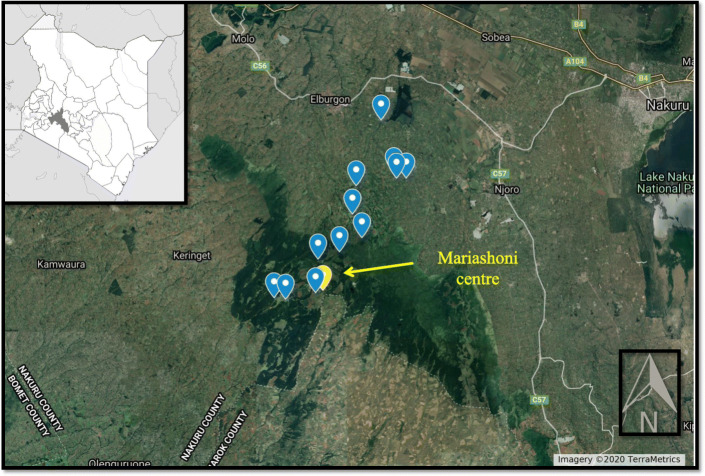
Map showing the study area and the visited localities (GPS points were recorded during the fieldwork) (File credits: Creative Commons Attribution-Share Alike 3.0 license)

Fieldwork was conducted between August 2019 and January 2020 among Ogiek communities living in the Eastern Mau Escarpment (Fig. 1). The study area is in the Mariashoni District, Molo Sub County, Nakuru, and covers 345.5 km^2^, extending from 2100 to 3000 m above sea level [49]. The major geomorphological features include escarpments, hills and plains. The area has a bimodal rainfall pattern, with rains between May and June and between September and November. Mean annual precipitation is 1200 mm and mean annual temperatures range from 12 to 16 °C, with the greatest diurnal variation occurring during dry seasons [50]. The Eastern Mau Complex can be vertically divided into four ecological areas: an open bushy forest at the edge of the plains (up to 2100 m a.s.l.), a more dense forest with large evergreen, semi-deciduous and deciduous trees (2100–2600 m a.s.l.), an upper bamboo forest (higher than 2600 m a.s.l.) and open grassland (2800–3000 m a.s.l) [51].

Currently, the majority of the Ogiek in Kenya live in the Eastern Mau Escarpment, especially in the Mariashoni District. Of the 12,000 people currently living in the area (among them Kikuyu, Kipsigis and Nandi), 4000 are Ogiek [48]. Mariashoni village (S 0° 22′ 06′′ E 35° 49′ 28′′) is one of the most important trade and business centres in the Eastern Mau Forest region. The economy of the area is based on cash crop farming (maize, potatoes, peas and wheat), subsistence farming and livestock rearing (cattle, sheep and goats) [36]. The Ogiek of Mariashoni District live in small villages located at middle and lower altitudes (2400–2700 m a.s.l.), often far from the remaining primary forest [42].

While in the past honey was used for domestic consumption and informal exchanges, it has now become an important source of income for Ogiek families. Honey is traded locally and in the nearest towns, from where it is then transported to major cities such as Nakuru and Nairobi. Since 2012, Ogiek beekeepers have been part of the Mariashoni Community Development Community-Based Organization (MACODEV CBO), which brings together around 350 beekeepers organised into 12 self-help groups, living in the area of Mariashoni and in the surrounding villages and settlements [52, 53]. MACODEV CBO finds its origin within a wider context of cooperation that has involved the Kenyan Ministry of Agriculture and the Kenyan NGO Network for Ecofarming in Africa (NECOFA) since 2004, and later on two Italian NGOs (Manitese and Ethnorêma). The project has aimed at increasing honey production and marketing through the introduction of modern beehives and equipment, the organization of training activities and the creation of a refinery unit [52]. Eventually, in 2015, Slow Food Kenya and Slow Food International launched a project to promote Ogiek honey, preserve Ogiek cultural heritage and protect the Mau Forest [54].

### Ethnobotanical data collection

We used in-depth semi-structured interviews, guided field walks in apiaries and participant observation to collect ethnobotanical data on the local names of plants, their phenology, ecology and uses in beekeeping, as well as ethnographic information on beekeeping practices. We conducted interviews with 30 Ogiek beekeepers, all of them members of MACODEV CBO, and with 10 additional stakeholders (i.e. cooperative workers, non-Ogiek beekeepers, NGOs representatives). The sampling framework was designed in order to be representative of the local population of producers in terms of age, gender, residence and involvement in the honey sector.

The interviews investigated the diversity of plants used for beekeeping, their folk names, distribution and uses (e.g. as melliferous plants, to build hives, to attract bees, to smoke out bees when harvesting honey, to store honey). We also collected information about beekeeping methods and tools, technical aspects related to the type of hive used, location of the apiaries, criteria used for their establishment and management techniques. We paid particular attention to the differences between traditional and modern hives in association with knowledge about melliferous and other plants used for beekeeping. During the walk-in-the-woods approach [55], we accompanied beekeepers in the forest and at hive locations during two different seasonal periods (January and August) and asked them to mention, among the plants we encountered, each species relevant for beekeeping purposes.

Before each interview, informed consent was obtained from each interviewee, as recommended by the code of ethics of the International Society of Ethnobiology, and the rationale, aims, methods and expected outputs of the project were explained to the interviewees in advance [56]. Interviews were conducted in Swahili and Ogiek in the presence of at least one research assistant that translated to and from English, recorded with a digital voice recorder and then transcribed into English.

### Specimen collection and identification

For each plant species named by the interviewees, voucher specimens were collected and used as prompts in other interviews for the purposes of triangulation of the information. For some plants for which specimens were not available, probable identification was obtained by asking the interviewees to describe the plant and its habitat and by comparing the recorded folk names with the existing literature [57–59]. A vegetation survey was also conducted in 8 apiary sites located in different ecological zones. The locations of the apiaries were recorded with global positioning system (GPS) and are reported in Table 1. Purposive sampling was employed during guided field walks along forest transects and through buffer zones. Voucher specimens were deposited in the Herbarium (NAI) of the University of Nairobi.

**Table 1:** Localization of the surveyed apiaries (GPS points were recorded during the fieldwork)

	Localities	Altitude	GPS coordinates	Apiary
Site 1	Ndoswa	2480 m	S 0° 21′ 48.45524′′ E 35° 51′ 56.53972′′	Modern hives (KTBH and Langstroth) Traditional log hives
Site 2	Ndoswa	2520 m	S 0° 21′ 48.97373′′ E 35° 51′ 28.47342′′	Modern hives (KTBH and Langstroth) Traditional log hives
Site 3	Ndoswa	2553 m	S 0° 21′ 32.27474′′ E 35° 51′ 20.26846′′	Modern hives (KTBH and Langstroth) Traditional log hives
Site 4	Mariashoni	2649 m	S 0° 22′ 10.86279′′ E 35° 49′ 29.78768′′	Modern hives (KTBH and Langstroth) Traditional log hives
Site 5	Songwi	2835 m	S 0° 27′ 40.64338′′ E 35° 45′ 27.44520′′	Traditional log hives
Site 6	Kiptunga	2924 m	S 0° 27′ 18.66697′′ E 35° 47′ 51.39118′′	Modern hives (Langstroth)
Site 7	Kiptunga	2927 m	S 0° 27′ 25.90239′′ E 35° 47′ 29.69104′′	Traditional log hives
Site 8	Songwi	2938 m	S 0° 25′ 25.26289′′ E 35° 48′ 40.30319′′	Traditional log hives

Based on these specimens, species identification was made by the authors according to *Upland Kenya wild flowers and ferns*: *A flora of the flowers*, *ferns*, *grasses*, *and sedges of highland Kenya* [59] and *Kenya trees*, *shrubs* and *lianas* [57]. For botanical nomenclature, we followed the criteria set by the The Plant List Database [60]. Folk names were transliterated into the Latin alphabet with the help of a local research assistant fluent in Ogiek and English. Additionally, we consulted bibliographic sources to confirm species names and uses [57–59, 61, 62].

### Data analysis

The study is largely based on a qualitative analysis of the interviews conducted. In order to address the first research question about the social and spatial organization of beekeeping in the different ecological zones of the study area, results from the interviews were verified and triangulated with the analysis of the walk-in-the-woods approach, of the GPS localities of the apiaries and of the surrounding beekeeping-associated plant species. Data analysis for the second research question about the identity of plants and their uses combined qualitative data from the interviews with the botanical identification of the plants mentioned, as described above. The third research question was addressed by combining the analyses above in terms of triangulation of the main attributes of local ecosystems and their flora with the beekeeping practices employed in different locations and the ethnobotanical knowledge that underpins them. Interview transcripts were entered into NVivo qualitative data analysis software (version 12.5.0 - QSR International, Melbourne, Australia), and codes, concepts and categories were generated during the analysis [63]. All the data were organised and subsequently selected and condensed as tables. The relevance of the species was calculated based on the number of mentions by the interviewees. We lastly carried out a comparative analysis between uses and associated knowledge in modern and traditional beekeeping to understand how and in what circumstances Ogiek beekeepers rely on one system, the other or a combination thereof.

## Results

### Characteristics of the interviewees

Table 2 presents the socio-economic and demographic profiles of the beekeepers interviewed. Their ages range from 30 to 71 years old with an average age of 48.6; 26 were men and 4 were women. They live in the area surrounding Mariashoni (*n*. 12) and in the localities of Ndoswa (*n*. 8), Kiptunga (*n*. 7) and Songwi (*n*. 3). Besides beekeeping, all the interviewees carried out farming activities and livestock rearing both for household consumption and for the market. Five were also employed by the government and local NGOs.

**Table 2:** Sociodemographic characteristics of the interviewees

Number of beekeepers	30			
Place of residence	Mariashoni	Ndoswa	Kiptunga	Songwi
	12	8	7	3
Gender	Males		Females	
	26		4	
Age range	30–39	40–49		50 +
	9	5		16
Main economic activities	Beekeeping	Agriculture	Livestock husbandry	Formal employment
	30	30	30	5
Total number of beehives^a^	Traditional log hives		Modern hives	
	~ 500		~ 250	
Individual hives^b^	Traditional log hives		Modern hives	
	26		12	
Collectives hive^c^	Traditional log hives		Modern hives	
	19		30	

^a^We estimate the total number of beehives owned by the beekeepers drawing from the interviews

^b^Number of beekeepers individually owning log and modern hives

^c^Number of beekeepers collectively owning log and modern hives through membership to self-help groups

### Beehives, ownership and beekeeping systems

**Fig. 2:**
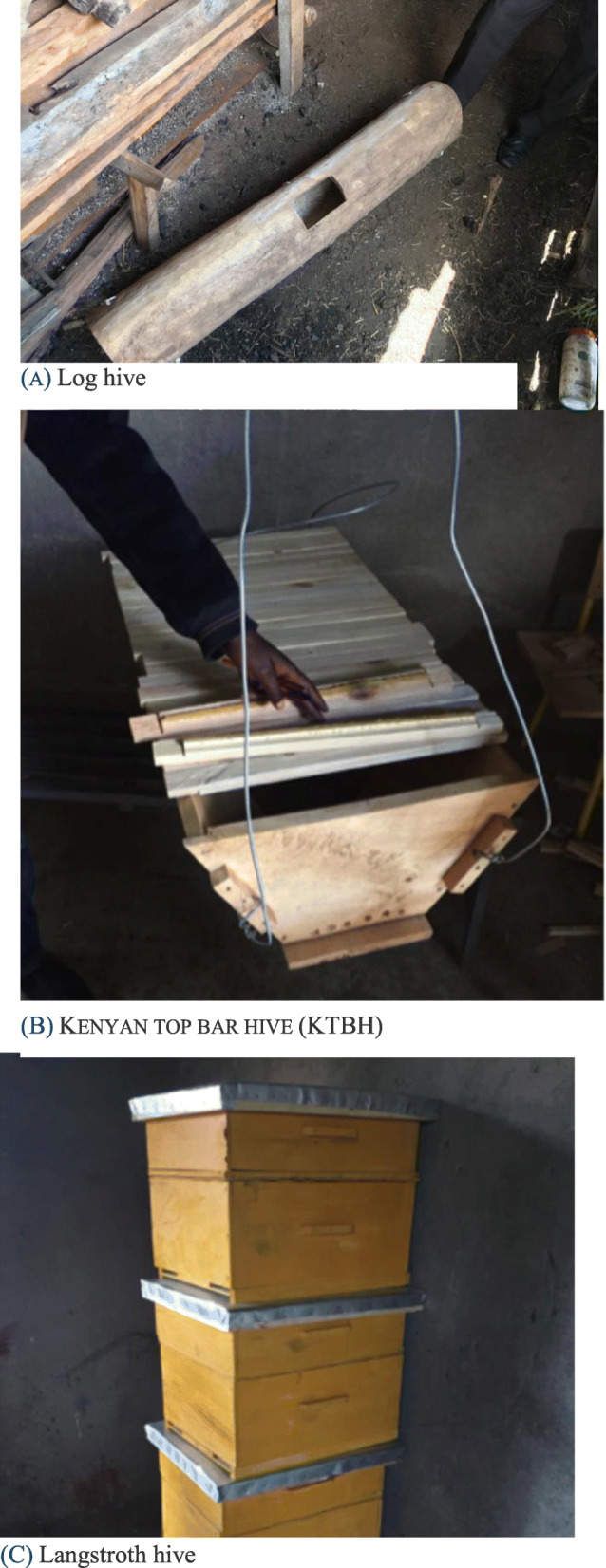
Beehives used by Ogiek beekeepers in the study area. Log hive (A), Kenya top bar hive (KTBH) (B), and Langstroth hive (C) (Photo: Dauro Mattia Zocchi)

An extensive beekeeping system based on traditional log hives and an intensive one based on modern beehives (KTB and Langstroth) coexist and intersect in Ogiek livelihoods. Three main types of beehives are used in the study area: (1) Log hives, which are made from hardwood trees and have fixed combs as in a wild colony; (2) Langstroth hives, which are the typical Western-style hive with movable frames; and (3) Kenyan top-bar (KTB) hives, which employ movable top bars rather than frames (Fig. 2). Modern and traditional hives are not used interchangeably but rather their use depends on several variables such as differential accessibility to hives and/or the material and skills to build them, intended ecological location, primary purpose (e.g. home-consumption, income generation) and social and cultural aspects (e.g. attachment to Ogiek cultural identity, food and medicinal properties of honey, etc.). Another important difference between log hives and modern hives relates to how they are obtained. While log hives are built by Ogiek beekeepers themselves on the basis of their traditional knowledge, most modern hives are gifted pre-made by NGOs and other organizations, involving little knowledge and skills on the side of beekeepers.

As shown in Table 2, beekeepers use both modern and traditional hives that can be owned individually or collectively by self-help groups. Some 70–75% of the total hives are traditional log hives, but their relative presence has been decreasing in favour of modern beehives in the last few years. Twenty-six beekeepers own individual log hives, inherited or built by themselves, while only 12 have individually owned modern hives, usually purchased from the market. In contrast, all the beekeepers collectively own modern hives that were gifted to the self-help groups by Necofa and other NGOs involved in the MACODEV CBO project. Nineteen beekeepers also have collective log hives that were either donated by members or purchased with the funds of the group.

Only four interviewees were women, and all of them owned beehives through the self-help groups to which each belongs, rather than individually. Beekeeping among the Ogiek remains a male-dominated activity, with some interviewees explaining that ‘women could not take part in honey harvesting due to the effort needed to climb the trees and reach the hives’ as well as for the fact that ‘they could not withstand the stings of bees like men.’ Nonetheless, the introduction of modern hives has facilitated women’s involvement in apiculture. Since modern hives are usually placed close to the ground and near the homestead, women can harvest and sell honey to the MACODEV cooperative through the group to which they belong.

### Honey bee species

Ogiek beekeepers distinguish two ‘kinds of bees’ colonizing their hives, namely ‘brown bees’ and ‘black bees’, which correspond respectively to *Apis mellifera scutellata* Lepeletier (the East African lowland honey bee) and *Apis mellifera monticola* Smith (the East African mountain honey bee) [64]. The former bees are described as having a brownish colour and being aggressive, while the latter as black and displaying ‘polite behaviour’. Brown bees are reported to be by far more common and more productive, while the honey from black bees is considered of better quality. No differences in the management of black and brown bees were reported in the interviews.

### Emic classification and vertical zoning

A customary emic classification of the forest in association with beekeeping emerges from the interviews. It includes three main zones that the beekeepers distinguish on the basis of altitude and vegetation type: Lower Forest (2300–2600 m), Central Forest (2600–2800 m), and Upper Forest (2800–3000 m). The main distinction is drawn between the Upper and Lower Forest, while the Central Forest is regarded more as a transition zone with features of the other two. While in the Upper Forest and to a lesser extent in the Central Forest beekeeping is tied to the presence of autochthonous species, in the Lower Forest the actual forest coverage has been largely replaced by exotic tree plantations and plots of cultivated land. Hence, what the Ogiek have traditionally called the Lower Forest is actually no longer a forest and the landscape is one of an agricultural frontier.

In the upper zone, beekeepers recognize a great diversity of trees and shrubs used in beekeeping. The species most often mentioned include *Nuxia congesta* R.Br. ex Fresen. *Dombeya torrida* (J.F.Gmel.) Bamps, *Podocarpus latifolius* (Thunb.) R.Br. ex Mirb. and *Ilex mitis* (L.) Radlk. On the edges of the Upper Forest, *Yushania alpina* (K.Schum.) W.C.Lin, *Micromeria imbricata* (Forssk.) C.Chr., *Trifolium burchellianum* Ser., *Lobelia bambuseti* R.E.Fr. & T.C.E.Fr., *Helichrysum argyranthum* O.Hoffm. and *Microglossa pyrifolia* (Lam.) Kuntze are the most common species.

The density and diversity of autochthonous species decrease with lower altitudes. In the cultivated lowlands, maize, bean, potato, sunflower, *Eucalyptus grandis* W.Hill., *Grevillea robusta* A.Cunn. ex R.Br. and *Cupressus lusitanica* Mill., all exotic and/or cultivated species, are the most valuable for beekeeping. However, interviewees also mentioned some forest species like *Dombeya torrida*, *Nuxia congesta*, *Trifolium burchellianum*, *Leonotis nepetifolia* (L.) R.Br. and *Polyscias kikuyuensis* Summerh., as individuals of these species still stand in the remaining patches of riverside forest and in the hedges of cultivated fields.

### Diversity of species and parts used in beekeeping

**Fig. 3:**
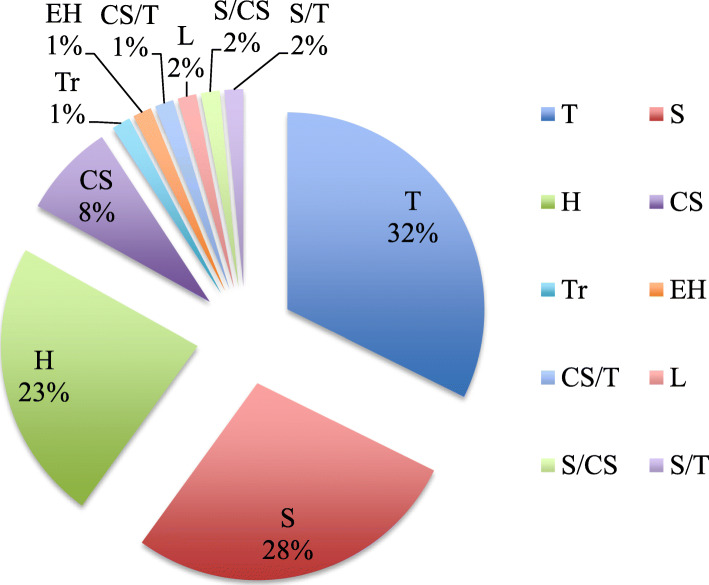
Distribution by growth habit of the 66 plant species recorded during the interviews. *T* tree, *Tr* tree trunk, *S* shrub, *S*/*CS* shrubs/climbing species, *S*/*T* shrubs/tree, *H* herb, *L* lichen, *EH* epiphytic herb, *CS* climbing species, *CS*/*T* climbing species/tree

The plant species mentioned in the interviews and used by the Ogiek for beekeeping purposes are listed in Table 3 in alphabetical order of botanical name. In total, 66 species (65 plants and 1 lichen) were recorded during the interviews. The species are distributed across 36 botanical families representing 58 genera. Asteraceae and Rosaceae are the most represented plant families with 9 and 6 species respectively, largely reported as melliferous. Fabaceae, Brassicaceae, Lamiaceae and Oleaceae are represented by 3 species each, while the remaining plant families by only one or two species. Trees are the most mentioned category of growth habit (32%), followed by shrubs (28%), herbs (23%) and climbing species (9%) (Fig. 3).

**Table 3:** Plant species mentioned by the interviewees as used for beekeeping purposes (in alphabetical order of botanical name)

Botanical name (voucher specimen code)	Family	Recorded folk name(s)^a^	Growth habit^b^	Part(s) mentioned	Use(s)	Frequency of citation^c^
*Abutilon mauritianum* (Jacq.) Medik. (DMZ2020/001)	Malvaceae	Goldoiywet (O)	S	Flower	Melliferous	+
*Achyranthes aspera* L. (DMZ2020/002)	Amaranthaceae	Sarurieet ap tisieet (O)	H	Flower	Melliferous	++
*Alchemilla* sp. (DMZ2020/003)	Rosaceae	Nyaek (O)^d^	H	Flower	Melliferous	++
*Allophylus abyssinicus* (Hochst.) Radlk. (DMZ2020/004)	Sapindaceae	Gipkosoriet/Maraisit (O)	T	Flower	Melliferous	+++
*Baccharoides lasiopus* (O.Hoffm.) H.Rob. (DMZ2020/005)	Asteraceae	Seregutiet (O)	S	Flower	Melliferous	++
*Brassica oleracea* L. (DMZ2020/006)	Brassicaceae	Mboga (S)/ Cabbage (E)	H	Flower	Melliferous (bees drink water from the leaves)	+
*Brassica oleracea* var. *viridis* L. (DMZ2020/007)	Brassicaceae	Sukuma wiki (S)	H	Flower	Melliferous	+
*Brassica rapa* L. (DMZ2020/008)	Brassicaceae	Mulo (O)	H	Flower	Melliferous	++
*Carduus nyassanus* subsp*. kikuyorum* (R.E.Fr.) C. Jeffrey (DMZ2020/009)	Asteraceae	Tegweyot (O)	H	Flower	Melliferous	++
*Carduus schimperi* Sch. Bip. (DMZ2020/010)	Asteraceae	Tegweyot (O) / Nyaek (O)^d^	H	Flower	Melliferous (source of pollen)	++
*Clematis simensis* Fresen. (DMZ2020/011)	Ranunculaceae	Pisinda (O)	CS	Bark	Ropes (ropes made from the woven fibres are used to fix the bark stripes around the log hives)	+
*Clutia abyssinica* Jaub. & Spach (DMZ2020/012)	Peraceae	Kiparnyat (O)	S	Flower	Melliferous	+
*Combretum molle* R.Br. ex G.Don (DMZ2020/013)	Combretaceae	Kemeliet (O)	T	Flower	Melliferous (source of pollen)	+
				Trunk	Placing hives (log hives are placed at the bifurcation of two branches strong enough to hold their weight; mostly in the lowlands)	+
*Crassocephalum montuosum* (S.Moore) Milne-Redh. (DMZ2020/014)	Asteraceae	Musumioit (O)	S	Flower	Melliferous	++
*Cupressus lusitanica* Mill. (DMZ2020/015)	Cupressaceae	Cypress (E)	T	Flower	Melliferous	+
				Trunk	Making hives (timber used mostly for modern beehives)	+++
*Cyathula cylindrica* Moq. (DMZ2020/016)	Amaranthaceae	Mutumiat (O)	CS	Flower	Melliferous	+
*Dombeya torrida* (J.F.Gmel.) Bamps (DMZ2020/017)	Malvaceae	Silibwet (O)	T	Flower	Melliferous (considered the best source of nectar, producing the most valued honey)	+++++
				Branches	Smoking hives (smoke from burning branches is blown inside the log hive to stun the bees before extracting the honeycomb)	+
					Attracting bees (branches are burnt inside the modern hives)	+
				Trunk	Making hives (the trunk is split in two longitudinally and used to build the log hive)	+
*Dovyalis abyssinica* (A.Rich.) Warb. (DMZ2020/018)	Salicaceae	Nukiat / Kigorwet (O)	T	Flower	Melliferous	++
*Eucalyptus grandis* W. Hill. (DMZ2020/019)	Myrtaceae	Eucalyptus / Blue gum (E)	T	Flower	Melliferous	++++
				Trunk	Making hives (timber used for modern beehives)	+
*Grevillea robusta* A.Cunn. ex R.Br. (DMZ2020/020)	Proteaceae	Gravelia (E)	T	Flower	Melliferous	++
				Trunk	Making hives (timber used mostly for modern beehives)	+
*Hagenia abyssinica* (Bruce ex Steud.) J.F.Gmel. (DMZ2020/021)	Rosaceae	Pontet (O)	T	Flower	Melliferous (source of pollen)	+
				Trunk	Placing hives (log hives are placed at the bifurcation of two branches strong enough to hold their weight)	++
*Helianthus annuus* L. (DMZ2020/022)	Asteraceae	Sunflower (E)	S	Flower	Melliferous	++
*Helichrysum argyranthum* O.Hoffm. (DMZ2020/023)	Asteraceae	Karabwet (O)	H	Flower	Melliferous	+++
*Hymenophyllum* sp. (DMZ2020/024)	Hymenophyllaceae	Susuot (O)	H	Flower	Melliferous	+
				Leaves	Harvesting tools (to clean hands and the leather bag after harvesting the honey)	++
					Harvesting tools (to cover the hole in the centre of the log hive from which honey is harvested)	+
*Hypoestes verticillaris* (L.f.) Sol. ex Roem. & Schult. (DMZ2020/025)	Acanthaceae	Nerubat netui (O)	H	Flower	Melliferous (source of pollen)	++
*Ilex mitis* (L.) Radlk. (DMZ2020/026)	Aquifoliaceae	Tongotwet (O)	T	Flower	Melliferous	++
				Trunk	Placing hives (log hives are placed at the bifurcation of two branches strong enough to hold their weight)	+
					Making hives (the trunk is split in two longitudinally and used to build the log hive)	+
*Jasminum abyssinicum* Hochst. ex DC. (DMZ2020/027)	Oleaceae	Mogoiywet (O)	CS	Flower	Melliferous	++
				Trunk	Placing hives (log hives are placed at the bifurcation of two branches strong enough to hold their weight; mostly in lowlands)	+
*Juniperus procera* Hochst. ex Endl. (DMZ2020/028)	Cupressaceae	Torokuet (O)	T	Bark	Covering hives (before hanging the log hive on tree, it is covered with bark stripes)	+++
					Attracting bees (pieces of dry bark are burnt inside modern hives)	+
					Smoking hives (to smoke traditional log hives along with *Usnea* sp*.* before extracting the honeycomb)	+++
				Branches	Storing honey (in the past honey was stored in a hollowed log of *Juniperus procera* placed one to few metres above the ground over a wooden frame in the forest)	++
				Trunk	Placing hives (log hives are placed at the bifurcation of two branches strong enough to hold their weight)	++
					Making hives (the trunk is split in two longitudinally and used to build the log hive; considered the best option for log hives)	++++
*Kniphofia thomsonii* Baker (DMZ2020/029)	Xanthorrhoeaceae	Yamyamt (O)	S	Flower	Melliferous (source of pollen)	++
*Leonotis nepetifolia* (L.) R.Br. (DMZ2020/030)	Lamiaceae	Mosipichiet (O)	S	Flower	Melliferous	++
*Lobelia bambuseti* R.E.Fr. & T.C.E.Fr. (DMZ2020/031)	Campanulaceae	Kabosuet (O)	S	Flower	Melliferous	+++
*Lobelia giberroa* Hemsl. (DMZ2020/032)	Campanulaceae	Tangaratwet (O)	S	Flower	Melliferous	++
*Microglossa pyrifolia* (Lam.) Kuntze (DMZ2020/033)	Asteraceae	Komereriet (O)	S	Flower	Melliferous	++
				Bark	Ropes (dry fibres are used to tie the two halves of the log hive)	+
					Storing honey (dry fibres of *Microglossa pyrifolia* and *Yushania alpina* are interwoven to make a bag where to store honey)	+
*Micromeria imbricata* (Forssk.) C.Chr. (DMZ2020/034)	Lamiaceae	Chepsagitiet (O)	H	Flower	Melliferous (source of pollen)	+
*Mikaniopsis bambuseti* (R.E.Fr.) C.Jeffrey (DMZ2020/035)	Asteraceae	Sereret (O)	CS	Flower	Melliferous	++
				Branches	Harvesting (beekeepers use the branches of the tree to climb the tree where the loghive is placed)	+
				Trunk	Placing hives (log hives are placed at the bifurcation of two branches strong enough to hold their weight; mostly in lowlands)	+
*Mimulopsis alpina* Chiov. (DMZ2020/036)	Acanthaceae	Sosonet (O)	S	Flower	Melliferous (flowering takes places every 10-12 years; when it happens, no circumcision ceremonies are held as it is a considered a bad omen)	++
*Musa × paradisiaca* L. (DMZ2020/037)	Musaceae	Ndizi (S) / Banana (E)	S	Flower	Melliferous	++
				Leaves	Covering hives (dry leaves are used to cover the traditional log hive before hanging it on trees)	+
					Storing honey (leaves used to make a basket used to transport and store honey)	+
*Nuxia congesta* R.Br. ex Fresen. (DMZ2020/038)	Stilbaceae	Choruet (O)	T	Flower	Melliferous (bees feed on it mainly during the rainy seasons)	+++
*Olea capensis* subsp*. macrocarpa* (C.H.Wright) I.Verd. (DMZ2020/039)	Oleaceae	Masaita (O)	T	Flower	Melliferous	+
				Trunk	Placing hives (log hives are placed at the bifurcation of two branches strong enough to hold their weight)	+
					Making hives (the trunk is split in two longitudinally and used to build the log hive; highly valued to build log hive)	+
*Olea europaea* subsp*. cuspidata* (Wall. & G.Don) Cif. (DMZ2020/040)	Oleaceae	Emitiot / Yemitioot (O)	T	Flower	Melliferous	+
				Trunk	Placing hives (log hives are placed at the bifurcation of two branches strong enough to hold their weight)	+
*Oxalis corniculata* L. (DMZ2020/041)	Oxalidaceae	Nyaek^d^	H	Flower	Melliferous	++
*Phaseolus vulgaris* L. (DMZ2020/042)	Fabaceae	Maragwe (S) / Bean (E)	H	Flower	Melliferous	+
*Pinus patula* Schltdl. & Cham. (DMZ2020/043)	Pinaceae	Pine (E)	T	Flower	Melliferous	+
				Trunk	Making hives (timber used mostly for modern beehives)	++
*Pittosporum viridiflorum* Sims (DMZ2020/044)	Pittosporaceae	Toponit (O)	T	Flower	Melliferous	++
				Trunk	Making hives (the trunk is split in two longitudinally and used to build the log hive; highly valued to build log hive )	+
*Plagiochila* sp*.* (DMZ2020/045)	Plagiochilaceae	Susuot (O)	Tr	Leaves	Harvesting tools (to clean hands and the leather bag after harvesting the honey)	++
					Harvesting tools (to cover the hole in the centre of the log hive from which honey is harvested)	+
*Plectranthus* sp*.* (DMZ2020/046)	Lamiaceae	Korpisiot (O)	H	Leaves	Harvesting tools (to clean hands and clothes after harvesting the honey)	+
*Podocarpus latifolius* (Thunb.) R.Br. ex Mirb. (DMZ2020/047)	Podocarpaceae	Saptet (O)	T	Bark	Covering the hive (before hanging the log hive on trees, it is covered with bark stripes)	+
				Trunk	Placing hives (log hives are placed at the bifurcation of two branches strong enough to hold their weight)	++
					Making hives (the trunk is split in two longitudinally and used to build the log hive; highly valued to build log hive )	++
*Polyscias kikuyuensis* Summerh. (DMZ2020/048)	Araliaceae	Ounet (O)	T	Flower	Melliferous	+
				Trunk	Placing hives (log hives are placed at the bifurcation of two branches strong enough to hold their weight)	+
					Making hives (the trunk is split in two longitudinally and used to build the log hive;highly valued to build log hive ; softwood)	++
*Prunus africana* (Hook.f.) Kalkman (DMZ2020/049)	Rosaceae	Tenduet (O)	T	Flower	Melliferous	+
				Bark	Covering hives (before hanging the log hive on trees, it is covered with bark stripes)	+
				Trunk	Placing hives (log hives are placed at the bifurcation of two branches strong enough to hold their weight)	++
					Making hives (the trunk is split in two longitudinally and used to build the log hive; highly valued to build log hive)	+
*Prunus* sp. (DMZ2020/050)	Rosaceae	Plum tree (E)	S	Flower	Melliferous	+
*Rapanea melanophloeos* (L.) Mez (DMZ2020/051)	Primulaceae	Korapariat (O)	T	Flower	Melliferous (source of pollen)	+
				Trunk	Placing hives (log hives are placed at the bifurcation of two branches strong enough to hold their weight)	+
*Rhoicissus tridentata* (L.f.) Wild & Drumm. (DMZ2020/052)	Vitaceae	Ingirenyit (O)	S	Flower	Melliferous	+
*Rubus pinnatus* Willd. (DMZ2020/053)	Rosaceae	Chepseonik (O)	S/CS	Flower	Melliferous	+
*Rubus steudneri* Schweinf. (DMZ2020/054)	Rosaceae	Taktakuet (O)	CS	Flower	Melliferous	++
*Schefflera volkensii* (Harms) Harms (DMZ2020/055)	Araliaceae	Chelumbut (O)	T	Flower	Melliferous	++
				Trunk	Placing hives (log hives are placed at the bifurcation of two branches strong enough to hold their weight)	+
*Scutia myrtina* (Burm.f.) Kurz (DMZ2020/056)	Rhamnaceae	Simbeywet (O)	CS/T	Flower	Melliferous	+++
*Searsia natalensis* (Bernh. ex C.Krauss) F.A.Barkley (DMZ2020/057)	Anacardiaceae	Sirondit (O)	S	Flower	Melliferous	+
				Trunk	Placing hives (modern and log hives are placed under this shrub’s canopy)	+
*Senna didymobotrya* (Fresen.) H.S.Irwin & Barneby (DMZ2020/058)	Fabaceae	Senetuet (O)	S	Flower	Melliferous	+
*Solanum nigrum* L. (DMZ2020/059)	Solanaceae	Managu (S)	H	Flower	Melliferous	+
*Solanum tuberosum* L. (DMZ2020/060)	Solanaceae	Viazi (S) / Potato (E)	H	Flower	Melliferous	++
*Syzygium cordatum* Hochst. ex Krauss (DMZ2020/061)	Myrtaceae	Lamaywet (O)	T	Flower	Melliferous	+
*Trifolium burchellianum* Ser. (DMZ2020/062)	Fabaceae	Dabibit / Puputiet / Nyaek (O)^d^	H	Flower	Melliferous (bees make propolis from it)	++
*Usnea* sp. (DMZ2020/063)	Parmeliaceae	Kurongurik (O)	L	Leaves	Smoking hives (dry lichens are burnt with the bark of *Juniperus procera* before harvesting the honeycomb)	+++
					Attracting bees (dry lichens are burnt inside modern hives)	+
*Vernonia auriculifera* Hiern (DMZ2020/064)	Asteraceae	Tepengwet (O)	S	Flower	Melliferous	++
				Branches	Smoking hives (smoke from burning branches is blown inside the log hive to stun the bees before extracting the honeycomb)	+
*Yushania alpina* (K.Schum.) W.C.Lin (DMZ2020/065)	Poaceae	Teegat (O)	S/T	Flower	Melliferous	++
				Leaves	Harvesting tools (to cover the hole in the centre of the log hive from which the honey is harvested)	+
				Branches	Smoking hives (dry branches are cut into small pieces and mixed with the branches of *Dombeya torrida* before harvesting the honeycomb)	+
				Trunk	Storing honey (mature trunks are cut and one side is covered with cow or sheep skin; the resulting container is used to transport and store honey)	++
*Zea mays* L. (DMZ2020/066)	Poaceae	Maindi (S) / Maize (E)	S	Flower	Melliferous	++++

For each species, we report the botanical name, botanical family, local plant name, growth habit, part(s) used, use in beekeeping and relevance (calculated based on the number of mentions by the interviewees)

^a^Recorded folk name(s): Ogiek, O; Swahili, S; English, E

^b^Growth habit: T, tree; Tr, Tree Trunk; S, shrub; S/CS, Shrubs / Climbing Species; S/T Shrubs / Tree; H, herb; L, lichen; EH, epiphytic herb; CS, climbing species; CS/T, Climbing Species/Tree

^c^Frequency of citation: +++++: mentioned by 70% of the informants or more; ++++: mentioned by 50% to 70% of the informants; +++: mentioned by 30% to 50% informants ++: mentioned by 10% to 30% of the informants; +: mentioned by less than 10% informants

^d^*Nyaek* is a collective name of several herbaceous species used as a source of nectar and pollen by bees

Regarding the plant parts most important for beekeeping purposes, flowers represent 60% of the total, followed by trunk (22%), leaves (7%), bark (6%) and branches (5%). The relative predominance of flowers reflects the fact that most of the species mentioned are melliferous plants used by bees as sources of nectar and pollen.

**Fig. 4:**
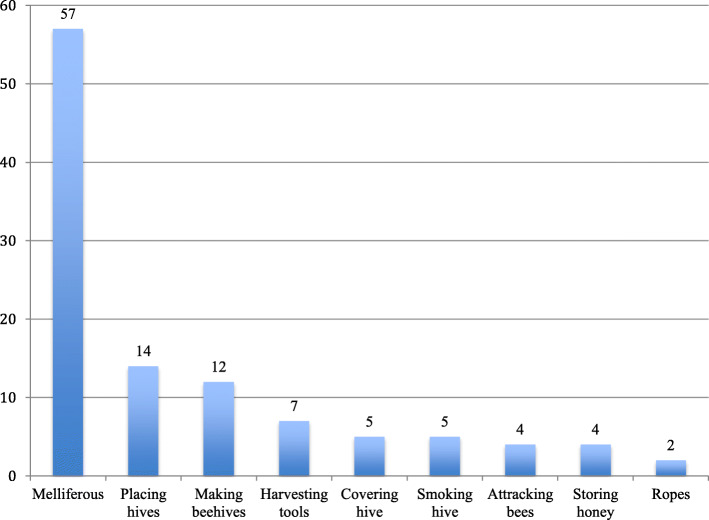
Means of use of the 66 plant species listed by the interviewees. Twenty-seven species had more than one use in beekeeping: 15 of them had two uses, 8 species had three uses, 3 species had four uses while only one species were used for six different purposes

As shown in Fig. 4, we identified nine different uses for the plant species mentioned, grouped into six main use categories, namely melliferous, making hives, placing hives, attracting bees, harvesting honey and storing honey. Twenty-seven species mentioned in the interviews have more than one use in beekeeping: 15 species have two uses, 8 have three uses, three have four uses, while one species, *Juniperus procera* Hochst. ex Endl., has six different uses. Overall, trees have a greater diversity of uses, as they provide pollen and nectar for bees and also materials for hive construction. The interviewees reported that for 12 species, the same part of the plant is used for more than one purpose.

### Melliferous species

Of the 66 species listed, more than 85% are melliferous, i.e. source of nectar (*n*. 50) or pollen (*n*. 7) for bees. Beekeepers distinguish between species used as a source of nectar or pollen by observing the behaviour of bees. In the latter case, ‘bees feed on the flower of these species but they do not produce honey’.

Malvaceae, Myrtaceae, Poaceae, Asteraceae, Rhamnaceae, Sapindaceae and Stilbaceae are the most mentioned families. Overall, the most salient melliferous species is *Dombeya torrida*, as 86.6% of the beekeepers considered its flowers as the best source of nectar in all the ecological zones. Bees feeding on it produce large amounts of honey that can be distinguished by its whitish colour and very sweet taste. Because of the long flowering period of the species, spanning from August to December [57, 62], *D*. *torrida* honey is valued as the main monofloral honey harvested in the area. On the other hand, about 30% of the interviewees mentioned *Carduus schimperi* Sch. Bip., *Combretum molle* R.Br. ex G.Don, *Hypoestes verticillaris* (L.f.) Sol. ex Roem. & Schult and *Kniphofia thomsonii* Baker as the most important species for pollen production.

The relative importance of melliferous species varies with the location of the beehives. In the Upper Forest, besides *D*. *torrida*, trees such as *Nuxia congesta*, *Allophylus abyssinicus* (Hochst.) Radlk. and *Ilex mitis* are the most mentioned. The importance attributed to these species stems from their central role in Ogiek ethnobotany. For instance, timber from *Ilex mitis* is used for the construction of traditional log hives and some species (e.g. *Allophylus abyssinicus* and *Nuxia congesta*) are also used for medicinal purposes as well as for cultural celebrations and traditional ceremonies (see also [30, 65]). In the cultivated lowlands, beekeepers instead highlighted the melliferous importance of blue gum (*Eucalyptus grandis*) and maize among exotic species and crops, while *Vernonia auriculifera* Hiern, *Baccharoides lasiopus* (O.Hoffm.) H.Rob., *Achyranthes aspera* L. and *Leonotis nepetifolia* were reported as the most valuable autochthonous species. These species are also abundant in disturbed areas in the Upper Forest [59].

### Making hives

Nineteen species provide raw materials for building hives. In particular, the Ogiek use timber from 12 tree species to build the structure of log hives (*n*. 8) and modern hives (*n*. 4), bark from 5 species to cover log hives before placing them on trees and fibres from one vine and one shrub are used as rope to tie together the two halves of the log hive and to affix the bark strips around it. The species most frequently used for hive making are *Juniperus procera* (50% to 70% interviewees), *Polyscias kikuyuensis* (10% to 30% interviewees) and *Podocarpus latifolius* (10% to 30% interviewees), whereas *Cupressus lusitanica* (30% to 50% interviewees) and *Pinus patula* Schltdl. & Cham. (10% to 30% interviewees) are the most common trees used to build modern beehives.

Traditionally, the process of log hive construction begins with a fallen tree, of the right species (i.e. *Juniperus procer*a), size and condition of decomposition (i.e. some decomposition facilitates working the trunk, while too much would endanger the hive’s resistance over time). Beekeepers split the trunk in two longitudinally, remove the bark and outer layers of wood and use the inner wood (reddish in colour) to build the hive. They hollow out the log with a smoother and leave the split hollow trunk to dry for at least 2 weeks. Subsequent steps involve tying the two halves together with a rope (*sagoet*) obtained from dry fibres of *Microglossa pyrifolia* and closing the two ends, leaving a small entrance for the bees at one end. Before hanging the hive, beekeepers cover it with bark strips that are then affixed to the hive with a cord made from the woven fibres of *Clematis brachiata* Thunb. Bark is mostly harvested from mature *Juniperus procera* trees, removing only small portions so as not to damage the trees. The majority of the beekeepers agree that hives made from *J*. *procera* are particularly resistant (lasting up to 10 years if managed properly) and warmer inside (with higher insulation capacity) compared to hives made from other trees, thus favouring bee occupation and persistence. However, the availability of fallen trees of this species has decreased in the last few decades due to forest logging and the replacement of autochthonous trees with exotic ones. Consequently, several beekeepers are replacing *J*. *procera* with softwood trees such as *Polyscias kikuyuensis*, *Podocarpus latifolius* and *Prunus africana*(Hook.f.) Kalkman. In the cultivated lowlands, where the density of autochthonous trees is low, some beekeepers (less than 10%) have replaced the former with exotic trees such as *Grevillea robusta* for the body of the hive, and dry leaves of banana trees to cover the log hive.

The construction of modern hives involves different practices, techniques and raw materials. Firstly, modern hives are built with timber from fresh cut exotic species such as *Cupressus lusitanica*, *Pinus patula* and, to a lesser extent, *Grevillea robusta*. According to Caroll [66], these are the most used and suitable timbers for the construction of modern beehives, especially KTB hives. However, the majority of the interviewees did not consider these trees, especially the timber of *C*. *lusitanica*, as the most appropriate for this purpose since timber from this species retains the cold and humidity inside the hive (more so when the timber is not dried properly), thus affecting bees’ activity and honey production. Also, as stated by one beekeeper, ‘bees are not used to these trees and do not feed on them (i.e. they are not a good source of nectar), they do not like their scent’. Some beekeepers using modern hives suggested addressing this problem either by building modern hives using autochthonous softwood species such as *Polyscias kikuyuensis*, *Podocarpus latifolius* and *Prunus africana*, or by covering the inner surface of modern hives with wooden panels from *Juniperus procera*.

Modern hives are covered with an iron sheet, a practice that has also been used for log hives. During some training sessions, one expert from a local agricultural college suggested this solution to beekeepers in order to cope with the scarce availability of bark from autochthonous trees. Those who tried they soon noticed a problem: the sound of the wind and the rain pounding against the sheet ‘scare’ the bees and induce them to abandon the hive.

### Placing hives and setting apiaries

Beekeepers mentioned 14 plant species (13 associated with the Upper Forest) used to hang log hives. They included 10 trees, one shrub and two vines, with *Juniperus procera*, *Podocarpus latifolius*, *Prunus africana* and *Hagenia abyssinica* (Bruce ex Steud.) J.F.Gmel. being the preferred species. As discussed above, these species are also the ones most mentioned as melliferous and as sources of timber and bark for hive construction, and are thus characterized by their multiple uses (6 different uses for *Juniperus procera* and 3 uses for *Prunus africana*, *Podocarpus latifolius* and *Hagenia abyssinica*). The degree of importance and availability of the tree species decrease with their decreasing density and changing ecologies from the upper to the lower areas. Thus, in the cultivated lowlands, beekeepers rely on species such as *Mikaniopsis bambuseti* (R.E.Fr.) C.Jeffrey, *Combretum molle* and *Jasminum abyssinicum* Hochst. ex DC to hang their hives.

**Fig. 5:**
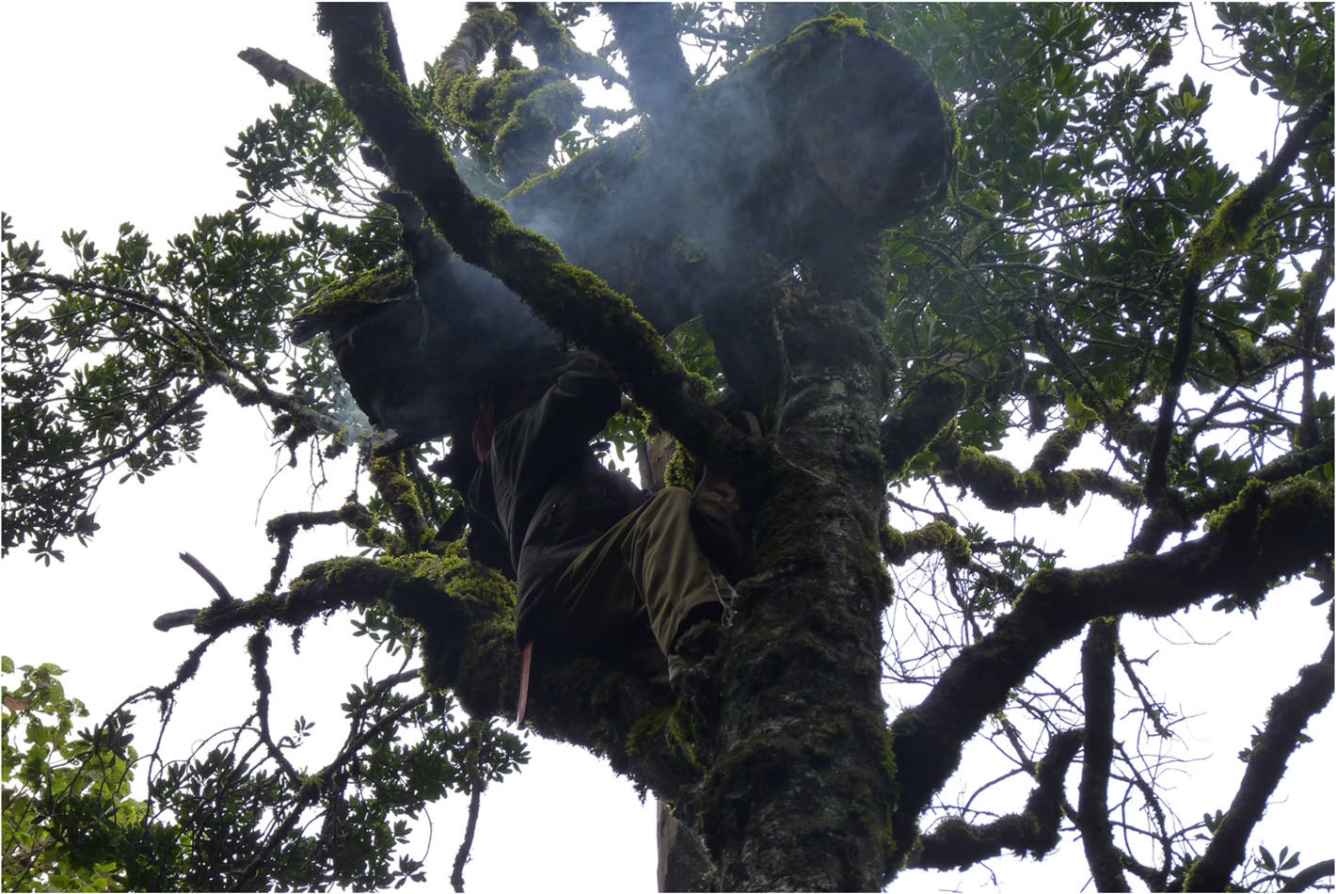
Traditional log hive hung on a tree in the forest (photo: Dauro Mattia Zocchi)

Because much of the spatial differentiation in the Mau landscape is vertical, with the agricultural frontier pushing into the forest from lower to higher altitudes, log hives predominate over modern hives at higher altitudes, and vice versa. In the Upper Forest, log hives are usually placed at a height of 5–10 m above the ground at the bifurcation of two branches strong enough to support their weight (Fig. 5). Other selection criteria include the density of branches (few branches make it difficult to climb the tree, whereas numerous branches expose the hives to raids by honey badgers and safari ants) and exposure to wind (hives are placed with the entrance facing downwind to avoid the cold and the sound of the wind beating against the hive). The density of melliferous species and distance to sources of disturbance for bees (e.g. livestock, people, wild animals, agricultural fields, etc.) are other variables considered in the hives’ positioning. Hives belonging to the same beekeeper are placed at a considerable distance from one another, possibly at different altitude levels, in order to best exploit the different blossoming seasons of different trees. The spreading of hives over a wide area may also function as a risk insurance mechanism, as, by doing so, beekeepers reduce the risk of catastrophic losses due to stochastic factors (e.g. theft, disease, etc.). In the Upper Forest area, we found only one collective apiary with modern hives. The Ogiek justify this on the basis of the difficulties in fitting modern hives, placed at ground level, to the forest ecosystem as a result of different factors, including adverse weather conditions, presence of predators (e.g. honey badgers, safari ants), disturbance by livestock grazing in the forest and difficulties in colonizing modern beehives.

**Fig. 6:**
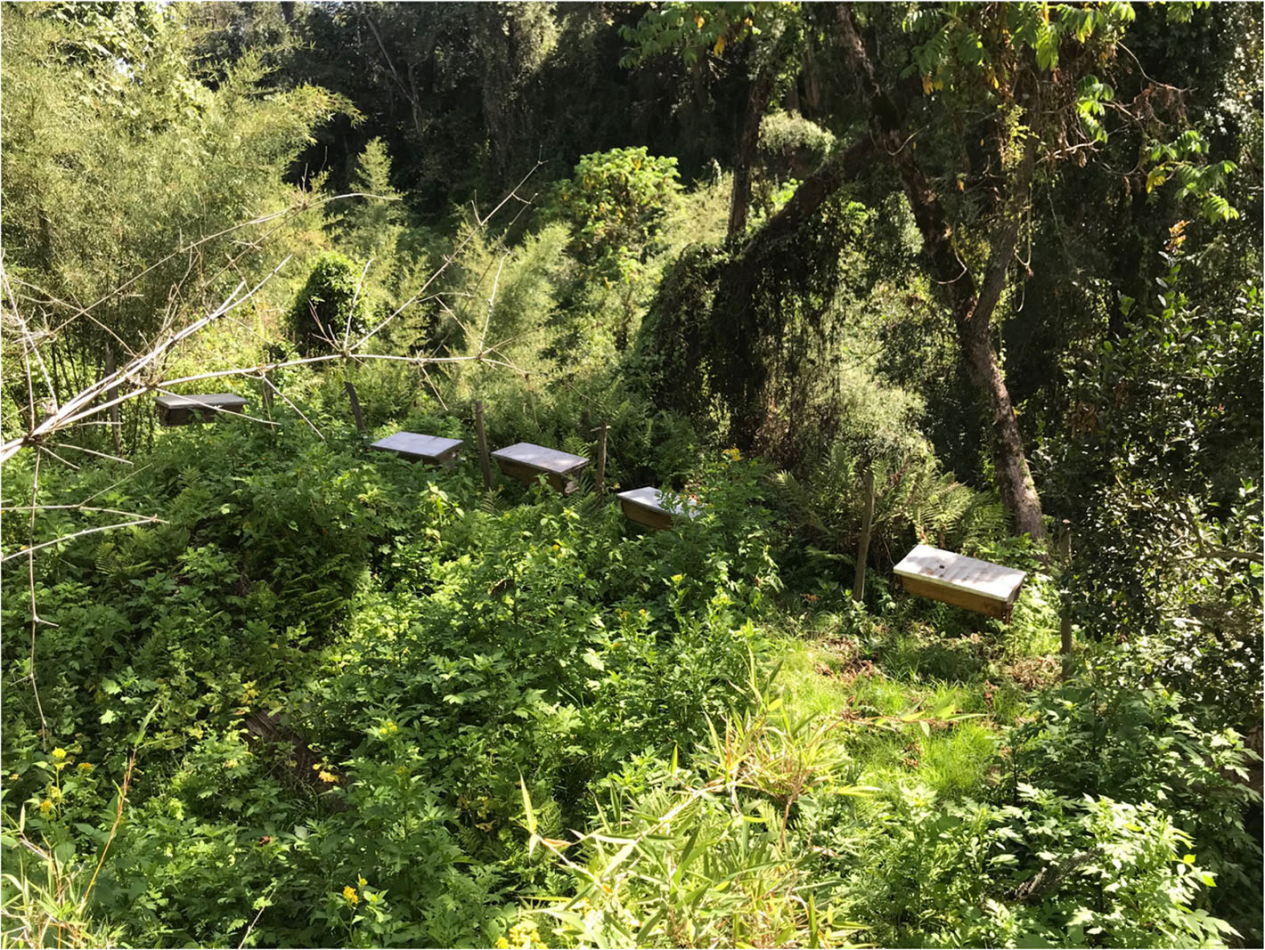
Modern apiary set in the lowlands (photo: Dauro Mattia Zocchi)

In the cultivated lowlands, modern beehives predominate. Apiaries are placed close to each other in open spaces (e.g. farmed fields, pastureland), preferably near the homestead, at the edge of forest patches, close to rivers, or a combination thereof, and in any case not far from crop fields (Fig. 6). In contrast to log hives, modern hives in the cultivated lowlands are grouped in fenced apiaries, including up to 40 hives in the same location. The majority of the beekeepers learnt these practices during workshops and training activities organised by NGOs and other actors involved in the MACODEV cooperative. Beekeepers did not mention relevant differences in the criteria used to set up apiaries of Langstroth and KTB hives. They are used interchangeably, and the relative presence of one or the other rather reflects the decisions of NGOs and extension offices on which kind to donate.

Some log hives are also present at lower altitudes, usually in the same apiary in which modern hives are located or in patches of forest close to homesteads. Some beekeepers moved a part of their log hives from the forest to the lowlands, close to their homesteads. In doing so, they avoid travelling long distances to reach their hives in the forest and they have more time for crop farming and livestock rearing. The changing ecology and the smaller size of trees in the lowlands compelled beekeepers to place their log hives closer to the ground (1–3 m high) or on a wooden base, surrounded or covered by shrubby vegetation that provides shade, nectar and pollen (e.g. *Searsia natalensis* (Bernh. ex C.Krauss) F.A.Barkley).

### Attracting bees

Traditional Ogiek beekeeping relies on swarming bees to spontaneously colonize a log hive. To facilitate this, beekeepers burn *Juniperus procera* bark inside the hive before placing it on a tree, thus impregnating it with its scent. Propolis is also burnt for the same purpose as well as smeared inside the hive to close cracks and better isolate it.

Occupation of modern hives, in contrast, marks a decreasing reliance on traditional knowledge and practices, and an intensification of human intervention in the process. Techniques employed include the use of catch boxes to trap swarms and a mixture of water and sugar to feed bees during dry periods. Knowledge of such practices does not stem from vertical intergenerational transmission but rather from training and workshops promoted by national and international organizations through the MACODEV cooperative. Only a few beekeepers (less than 10%) actually employ traditional methods to attract bees in modern beehives, as most others prefer to burn branches, bark and leaves of *Dombeya torrida* and dry lichen (*Usnea* sp*.*) and to smear the ashes on the inner walls. These two practices were not reported by the interviewees as being used in the Upper Forest with log hives (although the two plants are important for other purposes, i.e. as melliferous and for hive construction), and seem to be attempts of adapting traditional knowledge to modern beehive management.

### Harvesting and storing honey

**Fig. 7:**
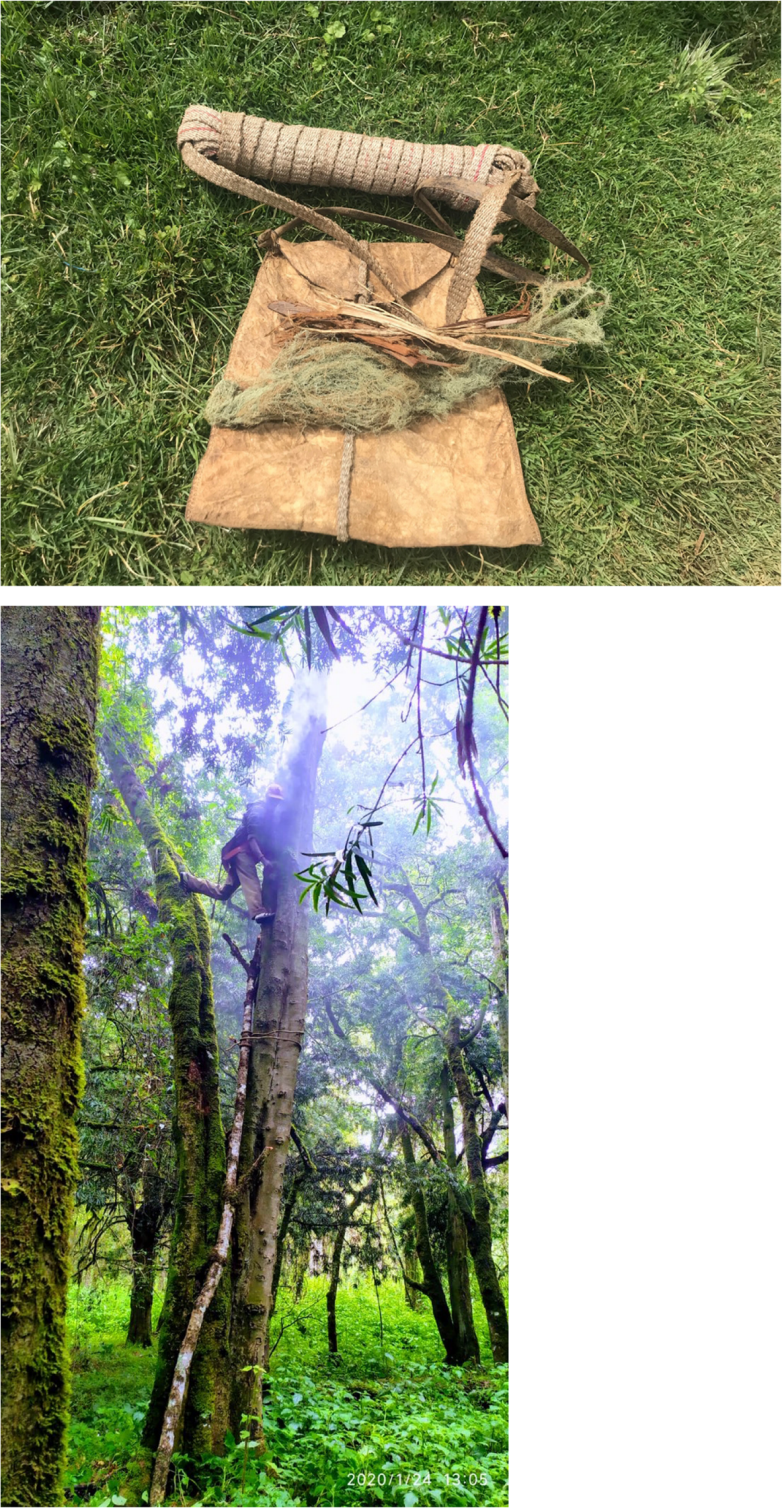
Traditional harvesting tools (**a**) and honey harvesting (**b**) (photo: Dauro Mattia Zocchi)

The interviewees mentioned seven species whose parts are used in 15 different ways to harvest and store honey. In the Upper Forest, beekeepers climb the tree and cut off the hive’s comb to harvest the honey. Besides a knife and a container, the beekeeper carries a bundle of green or dry lichen of the genus *Usnea*, locally known as *kurongurik*, which is burnt along with pieces of *J*. *procera* bark, called *sasiat*. *Sasiat* and *kurongurik* are carried inside the *motoget*, a bag made from the leather of cow or red duiker (*Cephalophus natalensis*) (Fig. 7). Honey is harvested by stunning the bees with smoke and extracting the honeycomb from a small opening on the bottom of the hive otherwise plugged with leaves of *Hymenophyllum* and *Plagiochila* species, both locally known as *susuot*.

Harvested honey is stored in two different containers, namely *soyet* and *poleita*, made, respectively, from *Yushania alpina* and *Microglossa pyrifolia*. The former, obtained by cutting a piece of bamboo and covering its ends with cow leather, is used to transport small quantities of honey as well as to harvest honey from stingless bees. The latter consists of a bag made from dry fibres in which honey is preserved for a long time. Another storing technique, used in the past but currently abandoned in favour of plastic buckets, involves keeping honey in a *kisungut*, i.e. a hollowed log of *Juniperus procera* sealed with propolis, covered with leaves and mosses, and placed one to a few metres above the ground over a wooden frame in the forest. The Ogiek stored that honey in the *kisungut* for years, consumed during times of food scarcity, especially by children, and used as a means of exchange with neighbouring communities [40, 48].

In the lowlands, with a limited presence of species such as *Juniperus procera*, *Dombeya torrida* and *Vernonia auriculifera* and a prevalence of modern hives, beekeepers use smoker guns, provided by NGOs and other organizations, fuelled by dry branches, leaves and sawdust, to stun bees, with no further mention of specific plants used.

## Discussion

### Ogiek ethnobotanical knowledge and beekeepers’ adaptation to a changing environment

Despite the three-fold customary division of the region into Upper, Central and Lower Forest, the analysis of beekeeping-associated ethnobotanical knowledge suggests that the Ogiek carry out beekeeping today distinguishing between two main zones: the Forest and the Lowlands. The former is the area still covered by primary Mau Forest, mostly concentrated in the Upper zone and dominated by trees such as *Prunus africana*, *Olea capensis* subsp. *macrocarpa* (Wall. & G.Don) Cif. and *Podocarpus latifolius*. The importance of trees and shrubs in the ethnobotanical knowledge of the Ogiek is in line with results from other studies on African beekeepers [3, 48, 62], and could be expected given the high reliance of Ogiek beekeeping on the forest both for melliferous species and for hive-construction purposes, as well as given the embeddedness of Ogiek traditional culture in the forest ecosystem and its main constitutive element, i.e. trees. In the forest, beekeeping has continued with traditional methods and is deeply embedded into the forest ecology and its species, and ethnobotanical knowledge is rich and detailed.

The Lowlands comprise the former Central and Lower Forest today engulfed by the agricultural frontier and represent a fragmented landscape where the primary forest has been replaced with open fields of cash crops, pastureland and exotic trees (conifer plantations of *Cupressus lusitanica*, *Pinus patula* and *Pinus radiata* D.Don and of *Eucalyptus* sp.), leaving behind only small forest patches [34, 36, 51]. Across the Lowlands, beekeeping is largely aimed at income generation and relies mainly on modern hives and methods sourced from external agents, and ethnobotanical knowledge encompasses exotic species and crops. Thus, while the Lowlands have become the interface through which the Ogiek adapt and reposition their culture and identity vis–à-vis the global beyond [47], the Forest maintains its role as the place of physical and cultural dwelling of log hives, a cyclical sense of time, the richest and most medicinal honey for home consumption. Although, due to the adoption of a more sedentary lifestyle [42], the Ogiek no longer base their livelihoods exclusively on the forest, they still have a strong material and cultural attachment to it and regard beekeeping and honey as main elements of their heritage.

Out of the 66 species identified during the ethnobotanical survey, 31 have been reported in earlier studies that explored the knowledge of Ogiek beekeepers with regard to the local flora [62, 63, 67, 68]. The vernacular names of 29 plants and their uses in beekeeping were also mentioned in ethnographic investigations carried out among Ogiek communities of the Mau Forest [37, 39, 68, 69]. While knowledge of bees and their preference for specific melliferous species is an important part of local ethnobotanical knowledge [62], Ogiek expertise also entails uses of plants for hive construction, placing hives, attracting bees, as well as harvesting and storing honey [48]. These bodies of knowledge are the result of a long and complex adaptation process of the Ogiek to the ecological settings of the Mau Forest, and they play an important role in the community’s resilience and cultural integrity.

However, due to the reduction of the primary forest surface and the restrictions of access imposed by the Kenyan government, Ogiek beekeepers are gradually losing their physical relationship with the Forest. While the beekeepers that are members of the Community Forest Association are allowed to access specific portions of the forest and use them for beekeeping and other activities [46], many live far from the Forest and are surrounded by cultivated fields, and progressively spend less time on forest beekeeping in favour of modern beekeeping in the Lowlands.

The ongoing initiatives of Ogiek honey promotion and beekeeping intensification are contributing to moving beekeeping literally and metaphorically out of the forest and into open areas of pasture and crop fields. In the process of adapting their honey-production system to the new ecological conditions of the cultivated lowlands, the Ogiek have begun placing new log hives close to their homesteads, relocating log hives from the forest to the lowlands and widely adopting modern beehives and associated techniques. These processes have led to the development of ethnobotanical knowledge about lowland and exotic species and about bees’ interactions with crop fields, as well as to a progressive integration of Ogiek beekeeping into commercial chains. While forest beekeeping depends on primary forest, the species therein and knowledge about them, modern beekeeping relies instead on external inputs (e.g. knowledge, materials) purchased in markets and shops (e.g. timber and tools to build hives) as well as obtained through relationships with extension services officers and international NGOs.

### Impacts of technological intensification on the ethnobotanical knowledge of the Ogiek and their relationship with the forest: between complementarity and hybridization

Beekeeping among the Ogiek appears to evolve through adaptive strategies that maintain a substantial distinction between traditional and modern beekeeping, between the Forest and the Lowlands. Indeed, the two sets of beekeeping practices largely demand different sets of knowledge, with an increasing relevance of external sources of knowledge with regard to modern hives and beekeeping in the Lowlands, and a persistence of vertical sources of ethnobotanical knowledge for what concerns log hives and beekeeping in the forest. The replacement of autochthonous trees by crops and exotic species reduces the possibility of carrying out beekeeping using traditional log hives, and a divergence between the two beekeeping systems therefore arises. This divergence expresses an overall complementarity of traditional and modern practices, a complementarity that is essential to expanding the reach of beekeeping beyond the limits of the Forest and into the cultivated Lowlands.

At the same time, both Ogiek beekeepers and external actors (e.g. NGO members, beekeeping experts, etc.) have recently explored ways to hybridize modern and traditional beekeeping systems. Particularly, beekeepers are devising ways to use autochthonous trees to build modern hives, and to promote bee colonisation of modern hives using traditional techniques. Through a trial and error process, beekeepers are modifying and adapting modern hives to the local ecological conditions, drawing from their understanding of bees and from their traditional knowledge. For instance, in the field of hive making, beekeepers are replacing the timber from *Juniperus procera* (which has become rare due to deforestation and logging) with timber from softwood trees such as *Polyscias kikuyuensis*, *Podocarpus latifolius* and *Prunus africana*, which were already used by the Ogiek in the past when the timber of *J. procera* was not available. At the same time, some attempts are ongoing to increase the productivity of traditional log hives with modern techniques (e.g. the introduction of a queen excluder) and to adapt log hives to the spatial and ecological conditions of the lowlands (e.g. replacing the covering bark with iron sheets or placing log hives on a wooden base rather than hanging them high on trees). These forms of incipient hybridization of beekeeping knowledge systems speak of the agency of the Ogiek concerning technological innovations and changes in the surrounding environment [29].

This study highlights that the choices of individuals and communities and their struggles should be understood within the wider framework of livelihood and community agency and adaptation to an environment in constant transformation [24, 28]. Through the adoption of modern beehives and the hybridization of modern and traditional knowledge and practices, the Ogiek have been able to carry on beekeeping in the cultivated lowlands, otherwise distant from the values and practices of their tradition, and to adapt and combine both traditional and externally-sourced knowledge to the new socio-economic and environmental conditions in which their livelihoods are embedded.

**Fig. 8:**
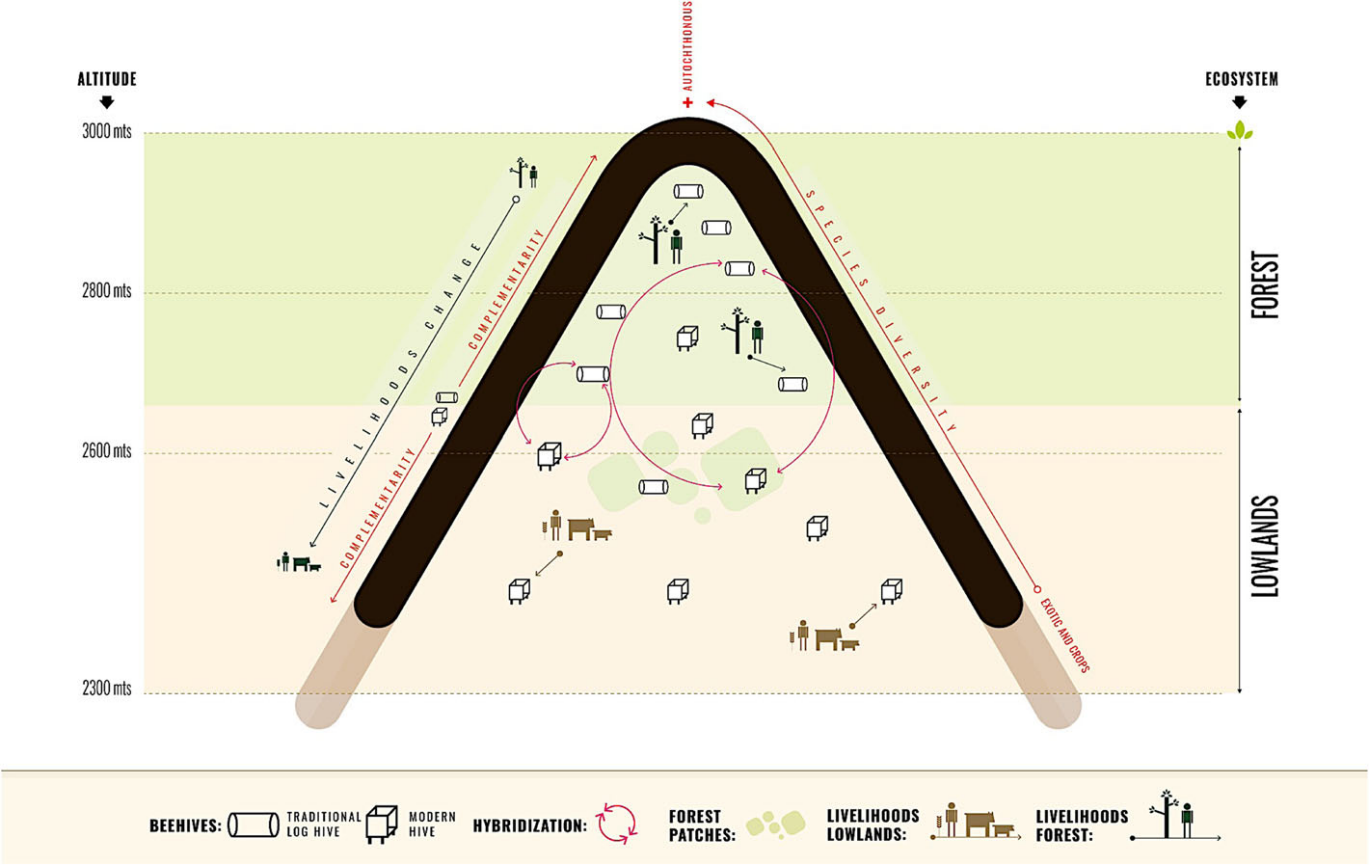
Complementarity and hybridization between traditional and modern beekeeping systems and livelihoods (Credit: Aarón Gómez Figueroa)

However, from the analytical lens of changing Ogiek-forest relations, the expansion of the reach of beekeeping in Ogiek livelihoods and toward the Lowlands can also be understood as a process of decoupling bees from trees and beekeeping from the forest, which reconfigures these relationships into open cultivated fields. Thus, while the advancement of agriculture is jeopardizing the very survival of the Mau Forest [36], the adoption of modern techniques is allowing the Ogiek to maintain honey production in ways that fit with the ecological characteristics of the agricultural frontier. Decoupling beekeeping and the forest, the introduction of modern hives and the relocation of some log hives close to agricultural fields make the forest dispensable in terms of honey production. Beekeeping becomes embedded into the spatial and social frame of the agricultural frontier, and this allows it to survive the forest with further frontier advancement. Figure 8 summarizes the dynamics of complementarity and hybridization between modern and traditional beekeeping systems.

As argued in other studies [18, 21], projects aimed at modernizing and intensifying beekeeping and other activities traditionally linked to forest environments risk ignoring the ecological, technological and socio-economic dynamics at play. The unfolding of these dynamics over time and space defines the trajectories that the social-ecological systems will follow in terms of forest conservation, household and community resilience and cultural integrity. While there is a general consensus regarding the positive externalities of beekeeping on the environment and rural livelihoods [2, 6], if the aspects mentioned above are not addressed, promotion and intensification initiatives may end up weakening traditional beekeeping and eroding the associated ethnobotanical knowledge.

## Conclusions

This study is an endeavour to understand the dynamics of livelihood and cultural change and adaptation associated with processes of promotion of local and indigenous products. Using Ogiek honey production as a case study, we have addressed the changes occurring in Ogiek beekeeping with the introduction of modern beehives, focusing on the associated ethnobotanical knowledge vis-à-vis changing livelihoods and ecological settings with the expansion of the agricultural frontier into the Mau Forest.

We have shown that modern and traditional beekeeping, rooted in different systems of knowledge and practices, are complementary within the livelihoods of the Ogiek largely in accordance with the ecological conditions in the selected apiary location: traditional in the forest, modern in the cultivated lowlands. However, this complementarity may conceal a contraposition of the two systems when seen through the lens of their relationships with the forest. Results suggest that the process of intensification of honey production based on modern beehives may have the potential to decouple beekeeping from the forest, weakening the continuity in the role of the forest in the livelihoods of the Ogiek and embedding honey, a traditional forest product, into the cultivated landscape of the agricultural frontier.

Between complementarity and contraposition, Ogiek beekeepers also experiment with hybrid forms of knowledge (e.g. by adapting traditional practices to modern design and vice versa). While processes of hybridization have the potential to further refine local beekeeping practices and increase honey production, more attention should be paid to log hive modernization and to the valorisation of forest honey as these could concurrently support the relationship between the Ogiek and the forest. As such, this research may be of relevance to policy makers, development institutions, and intervention programs that promote beekeeping in support of local livelihoods in forested areas of the tropics. Programs of intensification, valorisation and expansion of honey production through the introduction of modern beehives into traditional forest-based beekeeping systems have the potential to complement traditional honey production in the forest and expand the reach of beekeeping into the cultivated surroundings. But in order to avoid the risk of dissociating beekeeping from the forest and weakening the material and cultural links that tie indigenous populations to forest conservation, these programs should specifically target the continuity and resilience of forest beekeeping. This could be pursued through the promotion of log hive production and the valorisation of forest honey (e.g. differentiating forest honey as a high-quality product, narrating its importance and properties through informative labels) and that of the associated ethnobotanical knowledge and floristic diversity. A thorough understanding of traditional knowledge and the involvement of community members from the very start may help policy makers and development institutions to find endogenous elements that can be useful in the design of innovations supportive of beekeeper livelihoods and of the beekeepers’ role in forest conservation.

## Data Availability

Please contact author for data requests.
